# Dermal Health of Metal Machinists

**Published:** 2019-04

**Authors:** Mohammed JABBAR, Zailina HASHIM, Huda ZAINUDDIN, Rukman HAMAT, Munn Sann LYE

**Affiliations:** 1. Department of Environmental and Occupational Health, Faculty of Medicine and Health Sciences, University Putra Malaysia, Serdang, Malaysia; 2. Department of Population Medicine, Faculty of Medicine and Health Sciences, Universiti Tunku Abdul Rahman, Kajang, Malaysia; 3. Department of Community Medicine, Faculty of Medicine and Health Sciences, University Putra Malaysia, Serdang, Malaysia; 4. Department of Medical Microbiology and Parasitology, Faculty of Medicine and Health Sciences, University Putra Malaysia, Serdang, Malaysia

## Dear Editor-in-Chief

Metalworking fluid (MWF) is a complex mixture used during machining of metal objects and usually contains substances including biocides, corrosion inhibitors, metal fines, tramp oils, and biological contamination (1, 2). Water-based MWF causes much more skin disorders than oil-based fluids and irritant contact dermatitis is the main type of skin disorders (3).

A comparative cross-sectional study was conducted among metal machinists at Malaysia between 2014 and 2015 and focused on the dermal exposure to MWF and the consequences on dermal health of the workers in production section compared to workers in other work sections. The total participants of 398 workers who provided the consent to participate, 297 were randomly selected from production sections and 98 form other sections such as administration, financial, general store and others. The workers were exposed to MWF through direct dermal contacts.

The study was approved by the Ethics Committee for Research involving human subjects at Universiti Putra Malaysia (UPM).

The dermal exposure level to MWF was considerably higher among production sections workers than workers from other sections particularly on hands, forearms, and front torso (4). The common skin manifestations were redness, itchiness, scaling, or dryness (5).

Among the MWF exposed workers, those with dermal health disorders have showed poor compliances with occupational health and safety regulations such as wearing gloves, washing hands to the elbow at least 5 times per day, cleaning working zone (especially when open the gate of grinder machine to replace the grinding stone) and taking off the working uniform immediately after work (to prevent further dermal absorption of MWF from contaminated uniform). We recommend further studies to empower the workers to play a key role in protecting their health by increasing their awareness towards the health risks of exposure to MWF and to enhance their application of the above safety methods consistently and properly.
